# In Vitro Wound Healing Potential of Photobiomodulation Is Possibly Mediated by Its Stimulatory Effect on AKT Expression in Adipose-Derived Stem Cells

**DOI:** 10.1155/2021/6664627

**Published:** 2021-01-09

**Authors:** Naresh K. Rajendran, Nicolette N. Houreld, Heidi Abrahamse

**Affiliations:** Laser Research Centre, University of Johannesburg, Johannesburg 2028, South Africa

## Abstract

Increasing evidence suggests that adipose-derived stem cells (ADSCs) serve as a therapeutic approach for wound healing. The aim of this study was to determine the effect of photobiomodulation (PBM) on antioxidant enzymes in ADSCs. Four ADSC cell models, namely, normal, wounded, diabetic, and diabetic wounded, were irradiated with 660 nm (fluence of 5 J/cm^2^ and power density of 11.2 mW/cm^2^) or 830 nm (fluence of 5 J/cm^2^ and power density of 10.3 mW/cm^2^). Nonirradiated cells served as controls. Cell morphology and wound migration were determined using light microscopy. Cell viability was determined by the trypan blue exclusion assay. The enzyme-linked immunosorbent assay (ELISA) was used to measure the levels of antioxidants (superoxide dismutase (SOD), catalase (CAT), and heme oxygenase (HMOX1)). AKT activation and FOXO1 levels were determined by immunofluorescence and western blotting. The gaps (wound) in PBM-treated wounded and diabetic wounded cell models closed faster than the controls. PBM treatment significantly increased antioxidant levels in all cell models. This reflects that oxidative stress is reduced on the counterpart of increased antioxidant levels. This might be due to the activation of the AKT signaling pathway as evidenced by the increased AKT signals via western blotting and immunofluorescence. This data suggests that PBM promotes wound healing by increasing antioxidant levels by activating AKT signaling.

## 1. Introduction

Nonhealing chronic wounds are a prominent public health problem, affecting millions of patients worldwide [1]. Delayed healing of chronic wounds is characterized by a prolonged inflammatory phase, epigenetic changes, delayed cellular proliferation, poor reepithelialization, and impaired angiogenesis [2]. Wound healing in the skin is a complicated process that includes a series of biological events including cell proliferation and migration, reepithelialization, collagen deposition, and angiogenesis [3]. Because of limited cell sources, wound healing is adequately low in larger surface areas of skin wounds [4].

Increased blood sugar levels (hyperglycemic state) affect various phases of wound healing through different signaling mechanisms [5]. Hyperglycemia drastically increases the expression of proinflammatory cytokines, free radicals, and reactive oxygen species (ROS) [6], thereby increasing cellular oxidative stress. Under hyperglycemic conditions, an imbalance in ROS production and antioxidant levels leads to increased oxidative stress [7, 8]. High levels of ROS/reactive nitrogen species (RNS) promote oxidation of DNA, lipids, and proteins which results in cellular damage and cell death. Apart from cell death, increased ROS promotes cellular senescence by increasing protein denaturation, lipid peroxidation, DNA modification, and mitochondrial dysfunction [9, 10]. However, low quantities of ROS are required for various cell signaling and gene transcription processes, and only when there is an accumulation of intracellular ROS does it become deleterious to cells and leads to cell death [11]. Therefore, maintaining a normal redox balance is important for cell survival and growth.

Osteoblasts, chondrocytes, myocytes, adipocytes, and skin cells are all differentiated from mesenchymal stem cells (MSCs). MSCs are multipotent stromal cells [12]; therefore, these cells can be an optimal alternative source of cells that may be used for wound healing in the skin. Adipose-derived stem cells (ADSCs) are one of the many types of MSCs and are preferably used in dermatology [13]. A dominant source of MSCs is fat tissue, and the isolation of ADSCs is easy with a greater cell yield of 100–1,000 times compared to bone mesenchymal stem cells (BMSCs). ADSCs can participate in regeneration processes (through a paracrine effect, releasing cytokines, and growth factors) directly or indirectly [14]. Cherubino et al. found that the autologous transplantation of ADSCs showed an improvement in full thickness graft survival, scar reduction, and wound healing [15]. In addition, stem cells are capable of enhancing wound healing processes by inducing angiogenesis and stimulating collagen deposition by progressing alone or together with other growth factors [16].

Many preclinical studies reported the importance of ADSCs in regulating wound healing mostly by regulating matrix synthesis and deposition [17]. A study conducted by Shen and colleagues showed that treatment with artificial dermis and ADSCs with poly (L-glutamic acid)/chitosan scaffold (PLGA) increased new blood vessel formation and fibroblast proliferation that leads to the formation of a new dermis [18]. ADSC-treated wounds upregulate genes involved in matrix deposition that organize the cytoskeleton and provide a normal remodeling phase [19]. Combination therapy with growth factors and ADSCs can induce wound closure; however, the mechanism of growth factor-mediated wound healing processes is poorly understood.

Photobiomodulation (PBM) is considered a nonpharmacological, pain-free treatment option for diabetic foot ulcers, with positive effects [20–22]. Previous studies from our group have shown that irradiation of fibroblast cells (wounded cell model) grown under hyperglycemic conditions (diabetic wounded cells) and irradiated at 632.8 nm (23 mW) with either 5 or 16 J/cm^2^ showed increased proliferation and migration at a lower fluence of 5 J/cm^2^, while cells exposed to a higher fluence of 16 J/cm^2^ showed evidence of cell damage, decreased proliferation, and no cell migration [23]. It has also been shown that PBM at 636 nm (5 J/cm^2^, 95 mW) reduces apoptosis and inflammation (IL-1*β*), and at 660 nm (92.8 mW, 5 J/cm^2^), there is improved proliferation, migration, viability, and collagen type I in the same cells [24, 25]. Hypoxic cells irradiated at 636 nm showed an improvement in cellular morphology, an increase in proliferation, viability, and IL-6, and decreased apoptosis [26]. Both the wavelengths (660 and 830 nm) at a fluence of 5 J/cm^2^ have shown to be the most effective in stimulating WS1 human skin fibroblast cells, in both wounded and diabetic wounded cell models [23, 25]. Previous experiments on ADSCs have also shown that visible red (636 nm) and near-infrared light (825 nm) at a fluence of 5 J/cm^2^ stimulate the proliferation of these cells, and affected stem cell differentiation through modulation of cellular metabolism and redox status [27, 28]. Min et al. also showed that PBM at 830 nm enhances the proliferation and viability of ADSCs [29]. The objective of the present study was to determine the effects of PBM at 660 or 830 nm in regulating oxidative stress in ADSCs in vitro with applications in diabetic wound healing.

## 2. Materials and Methods

### 2.1. Cell Culture

This study was carried out on adipose-derived stem cells (ADSCs) (ATCC® SCRC-4000TM) and received ethical clearance from the Faculty of Health Sciences, University of Johannesburg, Research Ethics Committee (REC-01-28-2017). Four cell models, namely, normal (N), wounded (W), diabetic (D), and diabetic wounded (DW), were used. A diabetic model was achieved by growing the cells in complete media with an additional 17 mM/L D-glucose for more than two weeks to create a hyperglycemic environment [23, 25]. A “wound” was created on the cell monolayer prior to cell irradiation by using a sterile 1 mL pipette followed by a 30 min incubation at 37°C, 85% humidity, and 5% CO_2_ before irradiation [30].

### 2.2. Laser Irradiation

A continuous wave diode laser transmitting at a wavelength of 660 nm (Fremont, California, USA, RGBlase, TECIRL-70G-650SMA) with a power output of 102 mW or at 830 nm (Fremont, California, RGBlase, TECIRL-70G-830) with a power output of 94 mW was used for irradiation. The laser beam was directed from above towards cells via a fibre optic and had a spot size of 9.1 cm^2^, thus producing a power density of 11.2 mW/cm^2^ for 660 nm and a power density of 10.33 mW/cm^2^ for 830 nm. In order to irradiate cells with a fluence of 5 J/cm^2^, cells were irradiated in the dark for 446 s (45.39 J for 660 nm) or 484 s (45.59 J for 830 nm), respectively, in 3.4 cm^2^ diameter culture dishes containing 6 × 10^5^ cells in 1 mL fresh culture media. Postirradiation, cells were incubated for 24 h after which cell viability, nuclear damage, and expression of AKT, FOXO1, and enzymic antioxidants (SOD, CAT, and HMOX1) were determined. Nonirradiated cells served as controls (0 J/cm^2^).

### 2.3. Cell Viability and Migration

Cell viability was determined by the trypan blue exclusion assay in all cell models using 0.4% trypan blue (T8154, Sigma-Aldrich, South Africa). About 10 *μ*L of trypan blue and 10 *μ*L of cells were mixed and counted on the Invitrogen Countess™ II FL Automated Cell Counter. Migration of ADSCs in wounded models with/without laser irradiation was captured over a 48 h period using an inverted light microscopy and the AnalySIS getIT software (Olympus CKX41SF, Japan).

### 2.4. Hoechst Staining

Nuclear damage was determined by the Hoechst stain. Briefly, 6 × 10^5^ cells were cultured on sterile cover slips in 3.4 cm diameter culture dishes and incubated overnight, after which cells were irradiated with PBM at 660 or 830 nm. Twenty-four hours after laser irradiation, cells were incubated at room temperature with 1 *μ*g/mL Hoechst 33258 (H21491, Invitrogen, Thermo Fisher Scientific, South Africa) for 15 min. The fluorescence signals were examined using a Carl Zeiss Axio Observer Z1 (352Ex/461Em).

### 2.5. Enzyme-Linked Immunosorbent Assay (ELISA)

ELISA was used to determine enzymic antioxidants SOD, CAT, and HMOX1 in cultured cells. Cells were detached 24 h postirradiation (PBM at 660 or 830 nm) using TrypLE™ Select (12563-029, Gibco, Thermo Fisher Scientific, South Africa), and 3 × 10^4^ cells in 100 *μ*L media were seeded into 96-well microplates (Corning®, Costar® 3596, Sigma-Aldrich, South Africa) and allowed to attach. Cells were fixed in 8% paraformaldehyde for 15 min at room temperature and washed three times with PBS. Cells were permeabilized with 1% Triton X-100 in PBS for 30 min at room temperature and blocked in 2% blocking solution (10% bovine serum albumin (BSA) in PBS) for 2 h at room temperature. Plates were washed and incubated for 2 h with 1 : 50 primary antibody (mouse monoclonal IgG; Santa Cruz Biotechnology, SOD-1: sc-101523; CAT: sc-271358; HMOX1: sc-136960; Anatech Instruments (Pty) Ltd., South Africa). Plates were washed three times and incubated for 2 h with 1 : 5,000 secondary antibody (goat anti-mouse IgG-HRP, Santa Cruz Biotechnology, sc-2005, Anatech Instruments (Pty) Ltd., South Africa). Following rinsing, plates were incubated for 20 min with 3,3′,5,5′-tetramethylbenzidine (TMB) substrate and the reaction stopped with 1 M hydrochloric acid. Absorbance was read at 450 nm (PerkinElmer, Victor3™ multiplate reader).

### 2.6. Immunofluorescence

To examine the presence of AKT and FOXO1, 6 × 10^5^ cells were cultured on sterile cover slips for 12 h and irradiated at 660 or 830 nm. Cells were fixed 24 h postirradiation for 15 min in 4% formaldehyde at room temperature, washed twice in PBS, and permeabilized for 15 min at room temperature (0.01% *v*/*v* Triton X-100 in PBS). Cells were washed twice, blocked for 1 h (3% BSA in PBS), and incubated for 2 h at room temperature with 1 : 100 primary antibody (mouse monoclonal IgG; AKT: AHO-1112, Invitrogen, Thermo Fisher Scientific, South Africa; and rabbit monoclonal IgG; FOXO1: mAb-2880, Cell Signaling Technology, South Africa). The cells were washed twice and incubated for 2 h with 1 : 2,500 conjugated secondary antibody (goat anti-mouse FITC: Santa Cruz Biotechnology, sc-2010, Anatech Instruments (Pty) Ltd., South Africa). Nuclei were stained with 300 nM 4′,6-diamidino-2-phenylindole (DAPI; Invitrogen, D1306, Thermo Fisher Scientific, South Africa) and slides examined using the Carl Zeiss Axio Observer Z1 (FITC 494Ex/518Em and DAPI 358Ex/461Em).

### 2.7. Western Blotting

The Pierce™ BCA Protein Assay Kit (23225, Thermo Fisher Scientific, South Africa) was used to determine protein concentration. Cell lysates were diluted to 1 *μ*g/*μ*L in sample buffer (Laemmli 2× buffer; S3401, Sigma-Aldrich, South Africa) and heated at 110°C for 5 to 7 min. Protein samples (25 *μ*L) were separated by sodium dodecyl sulfate-polyacrylamide gel electrophoresis (SDS-PAGE) and transferred to an Immun-Blot PVDF membrane (162-0177, Bio-Rad, South Africa) for 3 h at 60 volts using a semidry blotter (B2529, Sigma-Aldrich, South Africa). Once the protein was transferred, 5% BSA in Tris-buffered saline (TBS) was used for blocking for 20 min. Membranes were incubated at 4°C overnight with primary antibody (mouse monoclonal IgG; AKT: AHO-01112, Thermo Fisher Scientific, South Africa; rabbit monoclonal IgG; FOXO1: mAb-2880, Cell Signaling Technology, South Africa; and GAPDH: sc-47724, Santa Cruz Biotechnology, Anatech Instruments (Pty) Ltd., South Africa). After washing three times for 10 min with 0.1% Tween-20 in TBS, membranes were incubated with horseradish peroxidase-conjugated secondary antibody (goat anti-mouse HRP: Santa Cruz Biotechnology, sc-2005, Anatech Instruments (Pty) Ltd., South Africa). After 2 h incubation, membranes were washed with TBS and incubated in the dark for 5 min with 1% 3,3′-diaminobenzidine (DAB) and 0.3% hydrogen peroxide in 5 mL PBS; developed bands were scanned. ImageJ software (National Institutes of Health, USA) was used for quantitative analysis of scanned densitometric values as ratios to GAPDH (used as a loading control).

### 2.8. Statistical Analysis

Data is represented as the standard error of the mean (SEM) of three repeats done in duplicate (*n* = 3). Statistical significance was analyzed using SigmaPlot version 13.0. Significant differences between irritated and control cells were determined by the Student *t*-test and between wavelengths using the one-way analysis of variance (ANOVA). Significant probabilities are shown as ^∗^*P* ≤ 0.05, ^∗∗^*P* ≤ 0.01, and ^∗∗∗^*P* ≤ 0.001.

## 3. Results

### 3.1. Cell Viability and Migration

**Figure 1:**
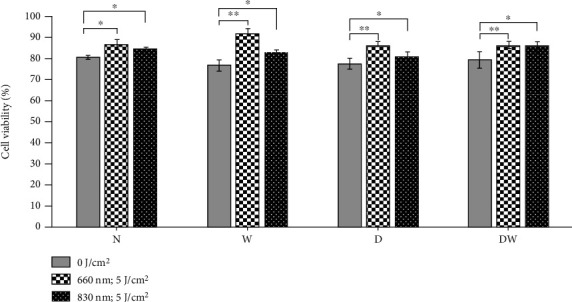
Effect of photobiomodulation on cell viability in normal (N), wounded (W), diabetic (D), and diabetic wounded (DW) ADSCs irradiated at a wavelength of 660 or 830 nm with a fluence of 5 J/cm^2^. Irradiated N, W, D, and DW cells showed a significant increase in cell viability as compared to nonirradiated cells. Data is expressed as the ±SEM (*n* = 3). Significant probability is shown as ^∗^*P* ≤ 0.05 and ^∗∗^*P* ≤ 0.01.

Figure 1 shows the viability of ADSCs in nonirradiated and irradiated normal (N), wounded (W), diabetic (D), and diabetic wounded (DW) models as determined by the trypan blue exclusion assay. A significant increase (*P* < 0.05) in cell viability was observed at both wavelengths in all irradiated cells. Comparison of the wavelengths showed that irradiation of W, D, and DW cells at 660 nm produced a significant increase (*P* < 0.05) as compared to the same cells irradiated at 830 nm.

**Figure 2:**
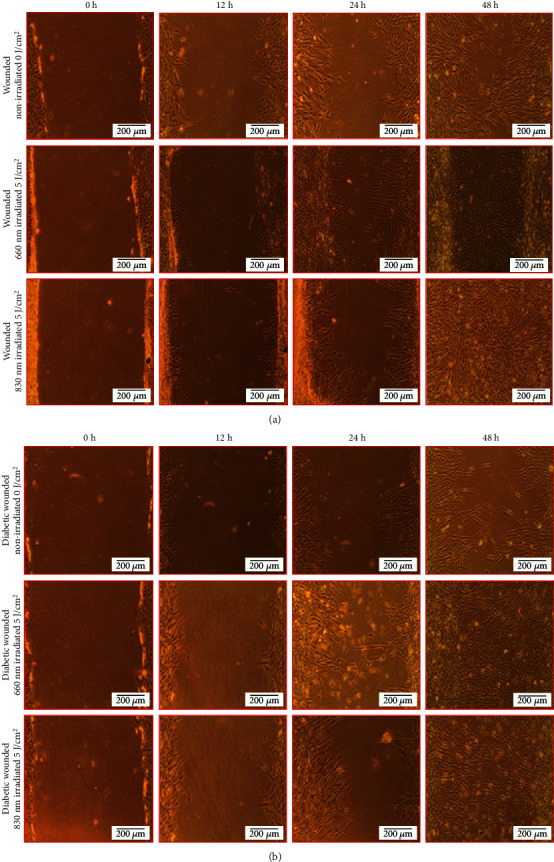
(a) Migration of ADSCs in a wounded cell model irradiated at a wavelength of 660 or 830 nm with a fluence of 5 J/cm^2^. Cells in the irradiated models migrated faster towards the center and more cells covered the central scratch at 48 h. (b) Migration of ADSCs in a diabetic wounded cell model irradiated at a wavelength of 660 or 830 nm with a fluence of 5 J/cm^2^. Cell migration in irradiated cell models was rapid and closed almost 90% of the scratch wound by 48 h.

Figures 2(a) and 2(b) show the morphology and migration of wounded and diabetic wounded ADSC models. Irradiated cells appeared normal in morphology (regular spindle-shaped structure) with increased cell migration towards the central scratch over time, and complete wound closure at 48 h. Nonirradiated models showed poor cell migration towards the central scratch and a delay in wound closure at 48 h.

### 3.2. Nuclear Damage Assessment by Hoechst Staining

**Figure 3:**
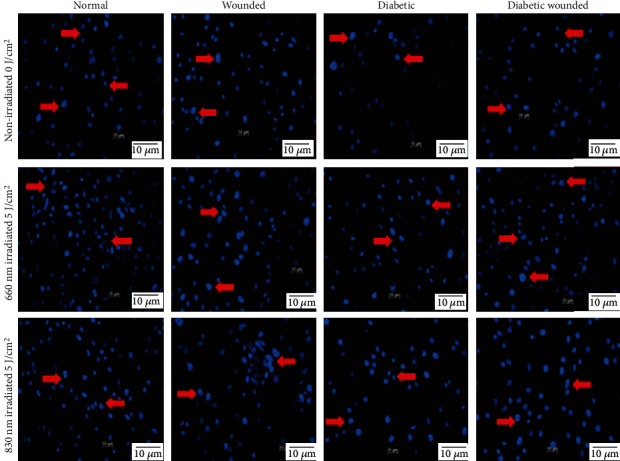
Nuclear damage assessment by Hoechst 33258 staining in ADSCs in normal, wounded, diabetic, and diabetic wounded cell models irradiated at a wavelength of 660 and 830 nm with a fluence of 5 J/cm^2^ and incubated for 24 h. In the nonirradiated groups, irregular-shaped nuclei, fragmented nuclei, and apoptotic cells (red arrow) were observed. In nonirradiated diabetic and diabetic wounded cells, there is an increase in the number of apoptotic cells and nuclear condensation. On the other hand, the irradiated groups showed uniformly stained nuclei, signifying dense nuclei without any fragmentation (red arrows). Magnification = ×100.

Nuclear damage in irradiated and nonirradiated cells was assessed 24 h postirradiation (Figure 3). Nonirradiated cells showed irregular-shaped nuclei with hollow centers and fragmented nuclei, whereas irradiated models showed clear spherical-shaped nuclei without any nuclear fragmentation.

### 3.3. AKT and FOXO1 Analysis

**Figure 4:**
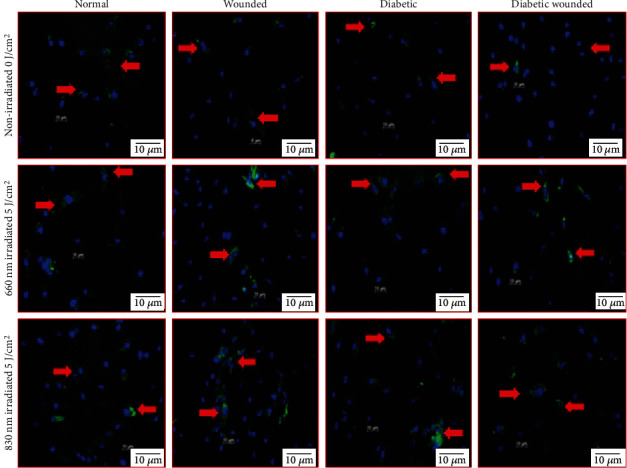
Effect of photobiomodulation on AKT in normal, wounded, diabetic, and diabetic wounded ADSCs irradiated at a wavelength of 660 and 830 nm with a fluence of 5 J/cm^2^. AKT is stained green with FITC, and nuclei are stained blue with DAPI. Both the irradiated and nonirradiated groups displayed positive staining for AKT proteins. Irradiated cells expressed more AKT protein compared to the nonirradiated groups. Red arrows indicate the presence of AKT. Magnification = ×100.

**Figure 5:**
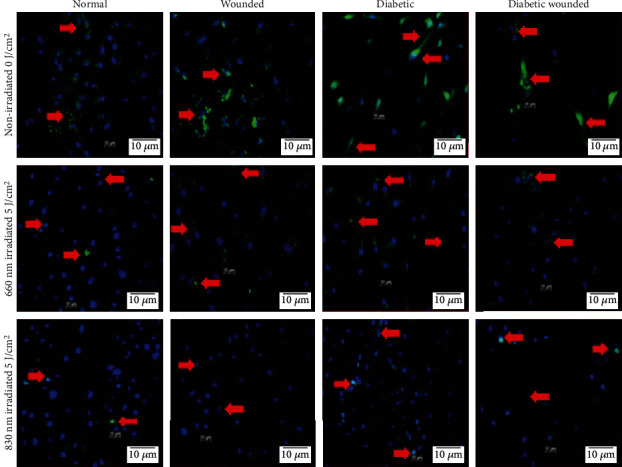
Effect of photobiomodulation on FOXO1 levels in normal, wounded, diabetic, and diabetic wounded ADSCs irradiated at a wavelength of 660 and 830 nm with a fluence of 5 J/cm^2^. FOXO1 is stained green with FITC, and nuclei are stained blue with DAPI. Both the irradiated and nonirradiated groups displayed positive staining for FOXO1 proteins. Irradiated cells expressed less FOXO1 compared to the nonirradiated groups. In the nonirradiated groups, there was an increase in nuclear translocation of FOXO1 in diabetic and diabetic wounded models compared to their normal counterparts. This is due to the increase in oxidative stress induced by growing cells under hyperglycemic conditions. Red arrows indicate the presence of FOXO1. Magnification = ×100.

**Figure 6:**
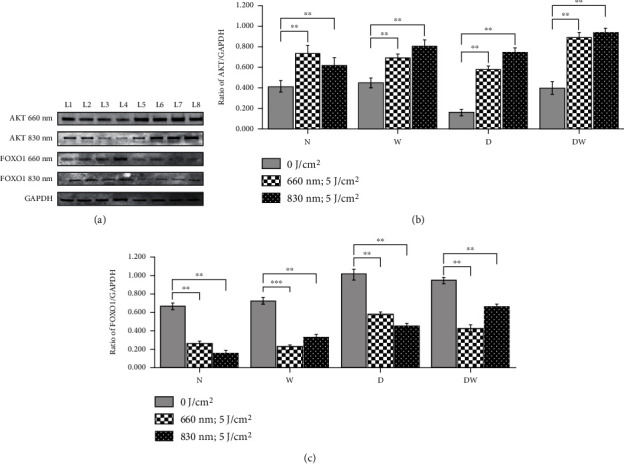
Effect of photobiomodulation on AKT and FOXO1 in normal (N), wounded (W), diabetic (D), and diabetic wounded (DW) ADSCs irradiated at a wavelength of 660 and 830 nm with a fluence of 5 J/cm^2^. GAPDH was used as a loading control. L1: normal (0 J/cm^2^); L2: wounded (0 J/cm^2^); L3: diabetic (0 J/cm^2^); L4: diabetic wounded (0 J/cm^2^); L5: normal (5 J/cm^2^); L6: wounded (5 J/cm^2^); L7: diabetic (5 J/cm^2^); and L8: diabetic wounded (5 J/cm^2^). (a) Representative blots. (b, c) Quantification of the ratio of the intensity of target protein divided by the loading control protein. Irradiated N, W, D, and DW cells showed a significant increase in AKT levels (b) and decreased FOXO1 levels (c) as compared to the nonirradiated cells. Data is expressed as the ±SEM (*n* = 3). Significant probabilities compared to the nonirradiated groups are shown as ^∗^*P* ≤ 0.05, ^∗∗^*P* ≤ 0.01, and ^∗∗∗^*P* ≤ 0.001.

Figures 4 and 5 represent the effect of PBM on AKT and FOXO1, respectively, by immunofluorescence. The increased oxidative stress under hyperglycemic conditions resulted in the diminished presence of AKT and increased presence of FOXO1 in diabetic and diabetic wounded cell models. Upon treatment with PBM at 660 or 830 nm, the fluorescent signal of AKT increased and FOXO1 decreased. Western blotting (Figure 6(b)) showed a significant increase in AKT levels in all irradiated cell models (*P* < 0.05). Comparison of the wavelengths showed that irradiation of N cells at 660 nm produced a significant increase in AKT levels (*P* < 0.01) as compared to the same cells irradiated at 830 nm, whereas W (*P* < 0.01), D (*P* < 0.01), and DW (*P* < 0.05) cells showed a significant decrease at 660 nm. Similarly Figure 6(c) shows a significant decrease (*P* < 0.01) in FOXO1 levels in all irradiated cell models. One-way ANOVA analysis comparison between wavelengths 660 nm and 830 nm showed that irradiation of N and D cell models at 660 nm showed a significant increase in FOXO1 levels (*P* < 0.05 and *P* < 0.01, respectively), whereas W (*P* < 0.05) and DW (*P* < 0.01) cells showed a significant decrease in FOXO1 levels at 660 nm compared to the same cells at 830 nm.

### 3.4. Expression of Enzymic Antioxidants

**Figure 7:**
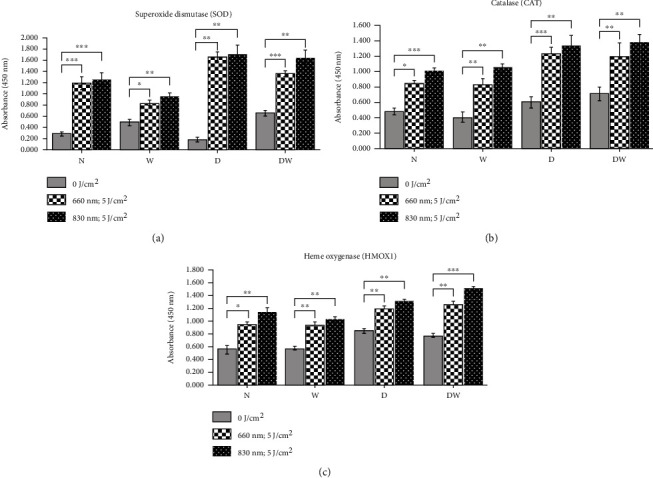
Effect of photobiomodulation on enzymic antioxidants in normal (N), wounded (W), diabetic (D), and diabetic wounded (DW) ADSCs irradiated at a wavelength of 660 and 830 nm with a fluence of 5 J/cm^2^. Data is expressed as the ±SEM (*n* = 3). Significant probabilities compared to the nonirradiated groups are shown as ^∗^*P* ≤ 0.05, ^∗∗^*P* ≤ 0.01, and ^∗∗∗^*P* ≤ 0.001.

Figure 7 shows the levels of enzymic antioxidants (SOD, CAT, and HMOX1) 24 h postirradiation. There was a significant increase in antioxidant levels in all the cell models (N, W, D, and DW) irradiated at 660 or 830 nm (*P* < 0.05). Comparison of the wavelengths showed that irradiation of N, W, D, and DW cells at 830 nm produced a significant increase in SOD (*P* < 0.05, *P* < 0.001, *P* < 0.05, and *P* < 0.01, respectively) and CAT (*P* < 0.01, *P* < 0.001, *P* < 0.05, and *P* < 0.001, respectively). HMOX1 levels in N, W, and DW cells irradiated at 830 nm were significantly increased compared to the same cells at 660 nm (*P* < 0.001, *P* < 0.05, and *P* < 0.001, respectively).

## 4. Discussion

PBM is a noninvasive method in which light at a particular wavelength and fluence is used to treat inflammatory diseases and stimulate tissue regeneration, resulting in the accelerated repair of chronic wounds [25, 31]. It has been shown that PBM effectively alters redox potential and intracellular ROS production [32]. Cell migration and proliferation are important for successful wound healing. Various studies have shown that PBM effectively stimulates cell proliferation and migration of fibroblasts and keratinocytes, as well as epithelial, endothelial, and mesenchymal cells. The wavelength range, fluence, and the time duration of irradiation influence the rate of migration and proliferation. Hawkins and Abrahamse found that PBM (at 632.8 nm with 2.5, 5, and 16 J/cm^2^) significantly increased human skin fibroblast (HSF) migration and proliferation. They also found that a lower fluence (2.5 and 5 J/cm^2^) is more effective than a higher fluence (16 J/cm^2^) in stimulating cell proliferation and wound healing. Many preclinical studies reported the importance of ADSCs in regulating wound healing, which is mostly through the regulation of angiogenesis and matrix formation [17]. Increased new vessel formation will enhance matrix deposition that will promote wound closure. Another study by Heo and colleagues showed that PBM at 660 nm with 3 J/cm^2^ attenuates oxidative stress by stimulating the expression of brain-derived neurotrophic factor (BDNF) via ERK and CREB signaling in the mouse hippocampus [33]. PBM (808 nm) induces the differentiation of human umbilical cord mesenchymal stem cells (hUC-MSCs) to neuronal cells and it also elevates nitric oxide to promote vasodilation [34].

PBM along with growth factors in complete media stimulates the differentiation of ADSCs into smooth muscle cells. Under normal conditions, PBM increases ROS generation, whereas under conditions of oxidative stress, PBM reduces ROS generation and apoptosis [35]. PBM treatment also stimulates wound healing in fibroblast cells by inhibiting ROS production and promotes cell proliferation by activating the ERK1/2 pathway to hasten wound closure [36]. In a similar study, irradiation of human skin fibroblasts at 660 or 830 nm with 5 J/cm^2^ showed that raised oxidative stress was reversed in diabetic and diabetic wounded fibroblast cell models, and there were increased levels of SOD, CAT, and HMOX1 [37]. Consistently, our experiments confirmed that PBM at 660/830 nm reduces diabetic-induced oxidative stress. The main mechanism of action of PBM is the absorption of photons by the respiratory chain mainly cytochrome c oxidase (by the heme and copper subunits). Once light gets absorbed, it upregulates mitochondrial membrane potential, cyclic AMPs, nitric oxide, and ROS that lead to the activation of various signaling pathways and transcription factors to stimulate the antioxidant defence system [38]. In this study, we found that PBM at 660 or 830 nm significantly increased the levels of the antioxidant enzymes (SOD, CAT, and HMOX1).

Our results showed that PBM at 660 and 830 nm significantly promotes wound closure by increasing ADSC migration towards the wound edge. Our results also showed a significant increase in cell viability, corresponding to the results observed in a similar study by de Villiers et al. [31], who observed an increase in cell viability in isolated ADSCs and a commercial ADSC cell line irradiated at a wavelength of 636 nm and a fluence of 5 J/cm^2^. Ebrahimpour-Malekshah et al. irradiated diabetic mice with an infected (MRSA) ischemic wound with or without transplanted ADSCs at 890 nm (power 80 W; pulse frequency 80 Hz; pulsed duration 180 ns; duration of exposure 200 s; power density 0.001 W/cm^2^; and energy density 0.2 J/cm^2^) 6 days per week for 16 days [39]. There was a significant decrease in microbial flora and inflammatory response and a significant increase in wound closure rate and strength and angiogenesis in all treated mice; however, it was found that PBM together with transplanted ADSCs showed superior results as compared to PBM and ADSC transplantation alone [39].

Wound healing is a well-organized sequential process that requires various cells, growth factors, cell-cell interactions, and signaling pathways. The AKT signaling pathway is important for cell survival, cell growth, cell proliferation/migration, angiogenesis, and regeneration. Similarly, Forkhead Box O1 (FOXO1) plays an important role in determining apoptosis and cell death and maintaining redox balance. An in vitro study using fibroblasts revealed that the knocking down of the FOXO1 gene increases cell proliferation and migration [40]. In diabetes, impaired healing is associated with the over expression of FOXO genes. In normal mice, FOXO1 promotes cutaneous wound healing by blocking oxidative stress through the TGF-*β*1 signaling pathway [41]. Mi et al. found that icariin promotes wound healing by increasing the migration and proliferation of keratinocytes by upregulating the AKT signaling pathway [42]. Our results show that PBM of ADSCs at 660 and 830 nm significantly increased AKT levels and attenuated FOXO1 levels, which hastened wound closure and enhanced the migration of cells, as was observed in the scratch wound assay. This was also evident in the diabetic model whereby ADSCs were grown under hyperglycemic conditions.

Changes in glucose metabolism may lead to tissue damage. Hyperglycemia increases the formation of free radicals, ROS, and advanced glycation end-products (AGEs) that directly or indirectly damage tissue and macromolecules. Once macromolecules (proteins, lipids, and DNA) are damaged, the normal cell cycle gets arrested which results in cell death. In our study, we found that the diabetic models showed fragmented/irregular-shaped nuclei suggesting cell death; however, this was not evident in irradiated cells, suggesting that PBM has a protective mechanism on these cells. Removal of free radicals/ROS and inhibition of AGEs may serve as a promising treatment for diabetic complications, e.g., antioxidant therapy well known for treating various diabetic complications. It is well known that a chronic hyperglycemic state is deleterious to cells and delays the wound healing process. This is due to an imbalance in the redox state, i.e., increased free radical production and reduced antioxidant activities. The oxidative stress condition promotes dysfunction of endothelial and smooth muscle cells that directly affect angiogenesis, thereby wound healing gets delayed. Antioxidants play an important role in scavenging free radicals and maintaining normal redox balance. Mi and colleagues [42] found that PBM at 632 nm with/without quercetin enhanced cutaneous wound healing in diabetic rats by elevating antioxidant levels and increasing the rate of migration/proliferation of fibroblasts, and accelerated matrix deposition [43]. Similar to this, our results have showed that PBM at 660 and 830 nm promotes in vitro wound healing by increasing the levels of antioxidants (SOD, CAT, and HMOX1) in ADSCs. When comparing the two wavelengths used in this study, a wavelength of 830 nm is more effective in attenuating oxidative stress. It was more effective in increasing AKT and antioxidant levels as compared to a wavelength of 660 nm. George et al. were also able to show that irradiation of ADSCs at 830 nm significantly upregulated the expression of the gene *SOD1*, along with other genes involved in redox mechanisms (*GPx*), glycolytic pathways (*HIF-1α*, *LDHB*, and *PKM2*), and mitochondrial activity (*TFAM*) [28].

## 5. Conclusions

In conclusion, this study has demonstrated that PBM can activate AKT signaling in ADSCs to promote wound healing under hyperglycemic conditions. Modulation of the AKT/FOXO1 signaling pathway might alter redox balance that contributes to delayed wound healing in diabetes. PBM effectively maintains normal redox balance and increases antioxidant levels by stimulating the AKT signaling pathways. From this study, it is clear that irradiation (660 and 830 nm with 5 J/cm^2^) is able to attenuate oxidative stress in ADSCs. However, a wavelength of 830 nm was more effective in attenuating oxidative stress in ADSCs, grown under physiological glucose concentrations and under hyperglycemic conditions than 660 nm.

## Data Availability

Data is available on request.
